# CXCL13 and neopterin concentrations in cerebrospinal fluid of patients with Lyme neuroborreliosis and other diseases that cause neuroinflammation

**DOI:** 10.1186/1742-2094-11-103

**Published:** 2014-06-11

**Authors:** Jukka Hytönen, Elisa Kortela, Matti Waris, Juha Puustinen, Jemiina Salo, Jarmo Oksi

**Affiliations:** 1 Department of Medical Microbiology and Immunology, University of Turku, Turku, Finland; 2 Department of Internal Medicine, Turku University Hospital, Turku, Finland; 3 Department of Virology, University of Turku, Turku, Finland; 4 Department of Neurology, University of Turku, Turku, Finland; 5 Division of Clinical Neurosciences, Turku University Hospital, Turku, Finland; 6 Department of Infectious Diseases, Division of Medicine, Turku University Hospital, Turku, Finland; 7 Faculty of Medicine, University of Turku, Turku, Finland

**Keywords:** *Borrelia burgdorferi*, Neuroborreliosis, CXCL13, Neopterin, Chemokine, Cerebrospinal fluid

## Abstract

**Background:**

Laboratory diagnosis of Lyme neuroborreliosis (LNB) is partly based on the detection of intrathecal *Borrelia burgdorferi*–specific antibody production (increased antibody index (AI)). However, AI can be negative in patients with early LNB and, conversely, can remain elevated for months after antibiotic treatment. Recent studies suggested that the chemokine CXCL13 in the cerebrospinal fluid (CSF) is a biomarker for active LNB. Also, CSF neopterin-level determination has been used to assess the degree of neuroinflammation in a wide variety of diseases.

**Methods:**

CXCL13 concentrations were analyzed in CSF samples of 366 retrospectively identified individuals. The samples represented pretreatment LNB (38 patients), non-LNB comparison patients, tick-borne encephalitis, central nervous system (CNS) varicella zoster virus infection, CNS herpes simplex virus infection, CNS HHV6 infection, CNS enterovirus infection, and untreated neurosyphilis. The panel included also samples from patients with multiple sclerosis and other neuroinflammatory conditions. Of the LNB patients, 24 posttreatment CSF samples were available for CXCL13 analysis. Neopterin concentrations were determined in a subset of these samples.

**Results:**

The CXCL13 concentrations in CSF samples of untreated LNB patients were significantly higher (median, 6,480 pg/ml) than the concentrations in the non-LNB group (median, <7.8 pg/ml), viral CNS infection samples (median, <7.8 pg/ml), or samples from patients with noninfectious neuroinflammatory conditions (median, <7.8 pg/ml). The use of cut-off 415 pg/ml led to a sensitivity of 100% and specificity of 99.7% for the diagnosis of LNB in these samples. CSF CXCL13 median concentrations declined significantly from 16,770 pg/ml before to 109 pg/ml after the treatment.

CSF neopterin concentration was significantly higher among the untreated LNB patients than in the non-LNB group. The use of neopterin concentration 10.6 n*M* as the cut-off led to a sensitivity of 88.6% and a specificity of 65.0% for the diagnosis of LNB. The CSF neopterin concentrations decreased statistically significantly with the treatment.

**Conclusions:**

These results clearly indicate that highly elevated CSF CXCL13 levels are strongly associated with untreated LNB. CXCL13 outperformed neopterin and appears to be an excellent biomarker in differentiating LNB from viral CNS infections and from other neuroinflammatory conditions.

## Background

Lyme neuroborreliosis (LNB) is a tick-borne infection of the nervous system caused by the *Borrelia burgdorferi sensu lato* spirochetes [1,2]. The diagnosis of LNB is based on a combination of anamnestic, clinical, and laboratory findings. According to the guidelines by European Federation of Neurological Societies, the laboratory diagnosis of LNB is based specifically on the detection of intrathecal *Borrelia burgdorferi*–specific antibody production (positive antibody index (AI)) [3]. Unfortunately, in some cases, the intrathecal antibody production is undetectable for several weeks after the onset of symptoms, leading to false-negative results. Conversely, AI may remain positive for months or even years after treatment, hampering the use of AI determination in later episodes of suspected LNB [4].

CXCL13 is a member of the CXC chemokine family. It selectively attracts B lymphocytes and helper T cells via the chemokine receptor CXCR5 [5]. In addition to being a chemokine involved in the development and maintenance of secondary lymphoid organs, CXCL13 is produced during inflammation also in nonlymphoid tissues, such as the central nervous system (CNS), where it functions as an attractor of B cells. Thus, it is understandable that CXCL13 levels in the CSF of LNB patients increase early in the course of the infection before any detectable antibody production, and CXCL13 in CSF has been suggested to be an early marker of LNB [6-12]. Also, the CXCL13 concentration in the CSF of LNB patients has been shown to decline rapidly after antibiotic treatment [6,13].

Neopterin is a pteridine derived from guanosine triphosphate. This proinflammatory factor is produced mainly by monocytes–macrophages and dendritic cells. Human endothelial cells and B-lymphocytes have been reported to be a source of neopterin. The proinflammatory cytokine interferon-γ is the main stimulus for neopterin production. Neopterin concentration in serum and CSF has been suggested to be a marker of disease activity in a wide range of infectious and inflammatory diseases [14-17].

The aim of the present study was to evaluate CXCL13 in comparison to neopterin as biomarkers for LNB and for monitoring response to therapy.

## Methods

### Patient samples

**Figure 1:**
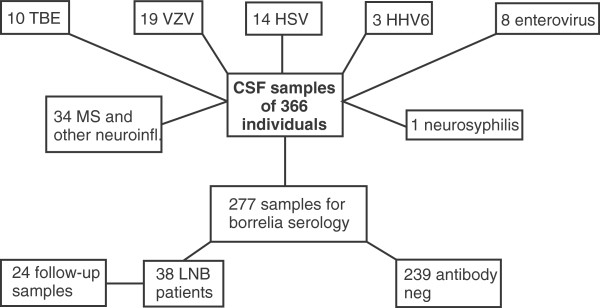
**An overview of CSF samples in which CXCL13 concentrations were analyzed in the study.** Neopterin levels were measured in 262 of the samples.

Patients were identified retrospectively from the laboratory information-management system of our laboratory. The patients were diagnosed according to the current Finnish guidelines, and the diagnoses were obtained from the patient records. All CSF samples were collected with informed consent from patients suspected to have a neurologic disease as a part of routine clinical practice. All samples were coded, and strict anonymity was maintained throughout the study. According to the Finnish Medical Research Act (No. 488/1999), Chapter 1, Sections 1, 2, and 3, the research in the present study is not medical research, and thus, it was not necessary to obtain a separate approval from the local Ethics Committee to use the samples in the assays of the present study. Altogether, CSF samples from 366 individuals were available for the study (Figure 1). After CSF-sample collection, the samples were sent at room temperature to our laboratory, where they were first stored at 4°C until borrelia serology was performed. After the serologic assays were completed, the samples were stored at −20°C until the chemokine analyses of the present study were initiated (length of storage from weeks up to 3 years).

LNB patients were diagnosed using the following criteria: 1) The patients had symptoms consistent with LNB, 2) other relevant diagnoses were excluded, 3) there was mononuclear pleocytosis in CSF of the patients with >5/ μl mononuclear cells and <100/ μl erythrocytes, 4) CSF samples were at a positive level for borrelia specific antibodies of IgM and/or IgG class as analysed by an in-house whole borrelia antigen (B. burgdorferi B31 sonicate) ELISA and/or with the commercial C6-peptide based assay (C6 Lyme ELISA; Immunetics Boston, Massachusetts, USA) using a 1:5 dilution of the CSF [18], and 5) intrathecal antibody production was observed as indicated by a positive (>0.3) IgM and/or IgG antibody index (AI) using the IDEIA Lyme Neuroborreliosis kit (Oxoid, Basingstoke, UK).

### Laboratory analyses

CXCL13 levels in 390 CSF samples (including 24 posttreatment LNB samples) were measured by using a human CXCL13 kit (Quantikine; R&D Systems, Minneapolis, MN, USA). The frozen samples were allowed to thaw at room temperature. The analyses were performed according to the instructions of the manufacturer. Samples with concentrations over the standard curve of the assay (>500 pg/ml) were further diluted 10 to 100 times with the calibration diluent buffer of the kit, and reanalyzed. Between the first analysis and the reanalysis, the samples were stored at −20°C.

Neopterin concentrations in CSF samples were measured by using Neopterin ELISA kit (IBL International GmbH, Hamburg, Germany) according to the instructions of the manufacturer. For neopterin-concentration analysis, 262 CSF samples (including 21 posttreatment LNB samples) were available.

Tick-borne encephalitis (TBE)-specific antibodies were measured by using SERION ELISA classic TBE Virus IgG/IgM kits (Institut Virion\Serion GmbH, Würzburg, Germany). For viral nucleic acid testing, total nucleic acid was extracted from CSF samples by using NucliSense easyMag extractor (BioMerieux, Boxtel, The Netherlands). Enterovirus RNA was detected with RT-PCR, as described previously [19]. DNA for human herpes viruses (varicella zoster (VZV), herpes simplex (HSV) −1 and −2, and human herpesvirus 6 (HHV-6)) was detected with individual qPCRs [20]. The performance of all of the PCR assays has been validated with appropriate proficiency-testing panels from Quality Control for Molecular Diagnostics (Glasgow, Scotland).

The single neurosyphilis case was diagnosed by using the following assays: Enzygnost Syphilis competitive enzyme immunoassay (Siemens Healthcare Diagnostics Products GmbH, Marburg, Germany) and *Treponema pallidum* hemagglutination assay (TPHA) (Cellognost-Syphilis H; Siemens) were used for the detection of treponemal antibodies, and VDRL (Venereal Disease Research Laboratory) carbon antigen agglutination test (Oxoid) was used to detect nontreponemal antibodies.

### Statistical analyses

Statistical analyses were performed by using IBM SPSS Statistics 22. To test for the normality of the data, the Kolmogorov-Smirnov test was applied. Differences in CXCL13 and neopterin concentrations between groups were analyzed by using the Kruskal-Wallis test followed by single pairwise comparisons with the Mann–Whitney *U* test and Bonferroni corrections. Wilcoxon signed-rank test was used to compare concentrations before and after treatment. Correlations were done by using Spearman rank-order correlations. *P* values of <0.05 were considered significant.

The CSF quotients of mononuclear cells, CXCL13, or neopterin before and after treatment among the sample pairs (where the posttreatment sample was collected at the end of the treatment) were calculated as follows: pretreatment CSF sample value divided by the posttreatment CSF sample value regarding CSF mononuclear cells, CXCL13, and neopterin concentrations.

## Results

CSF samples from 366 retrospectively identified individuals were available for the present study (Figure 1). Of these samples, 38 were borrelia antibody-positive pretreatment samples, of which 31 were from confirmed LNB patients. Seven patients did not fulfill all the LNB criteria presented in Materials and methods, and thus they represented probable LNB cases. AI was negative or not done in six of the probable patients, and in one patient, the CSF mononuclear cell count was below 5/μl. Of the 31 confirmed LNB patients, 24 posttreatment CSF samples were available for CXCL13, and 21, for neopterin determinations, respectively. Nineteen of the posttreatment samples available for CXCL13 analysis were collected 2 to 4 weeks after the pretreatment samples, whereas in five cases, the second sample was collected several months (108 to 223 days) after the first sample. For neopterin measurement, 16 posttreatment samples collected at the end of treatment and five samples collected months after the treatment were available. All 38 LNB patients were borrelia antibody positive in the serum, as determined by the in-house whole-borrelia antigen ELISA [21], and/or with the C6-peptide-based assay (C6 Lyme ELISA; Immunetics) (data not shown).

The presenting symptom in 30 of the LNB patients was radiculitis or pain radiating to the upper or lower limbs or to the trunk. Nineteen patients had facial nerve paralysis. Thirteen patients had both symptoms. In addition, some patients had intermittent or continuous headache, eye manifestations, dizziness, or paresis. The median duration of symptoms before the first CSF sample was 30 days, with the range from zero to 120 days. LNB patients were treated with either intravenous ceftriaxone, oral doxycycline, or with a combination of both of these drugs. The non-LNB group included 239 CSF samples that were sent to our laboratory for borrelia antibody analysis, and that were found to be borrelia antibody negative. Baseline characteristics of the LNB patients and the non-LNB subjects are listed in Table 1.

**Table 1:** Baseline characteristics of the untreated and treated LNB patients and of the borrelia antibody negative patients

				**Antibodies**									**Presenting symptom**	
**Group**	**No**	**Age, years (Median, range)**	**Sex (F/M)**	**B31**^**a **^**(pos/neg)**	**C6**^**b **^**(pos/neg)**	**IT-index**^**c **^**(pos/neg/ND)**	**Cells/μl**^**d **^**(Median, range)**	**OC-bands**^**e **^**(pos/neg)**	**Protein (mg/ml) (Median, range)**	**Duration of symptoms**^**f **^**(days) (Median, range)**	**Tick bite**^**g **^**(No of patients)**	**EM**^**h **^**(No of patients)**	**FP**^**i**^	**Radiculits/Radiating pain**
**Untreated LNB**	38	66 (3–80)	16/22	37/1	37/1	29/3/6	104 (0–1516)	21/4	1034 (454–3148)	30 (0–120)	11	2	19	30
**Treated LNB**	24	64 (34–79)	8/16	22/2	18/6	19/4/1	35 (0–120)	10/0	601 (267–1597)	NA^j^	NA	NA	NA	NA
**Non-LNB**	239	50 (1–91)	133/106	0/239	ND^k^	ND	NK^l^	NK	NK	NK	NK	NK	NK	NK

^a^CSF antibodies to *B. burgdorferi* s.s B31 whole cell antigen.

^b^CSF antibodies to C6-peptide.

^c^Intrathecal antibody production index.

^d^Cells/μl, mononuclear cell count.

^e^OC-bands, oligoclonal bands in CSF.

^f^Duration of symptoms before first CSF sampling.

^g^Observed tick bite.

^h^Observed erythema migrans.

^I^FP, facial nerve paralysis.

^j^NA: not applicable.

^k^ND: not done.

^l^NK: not known.

Samples for the comparison groups were selected based on the conception that the associated clinical manifestations are potential differential diagnostic options for LNB. The comparison samples from patients with other CNS infections included (a) ten CSF samples from patients with TBE, (b) 19 samples from patients with CNS VZV infection; (c) 14 samples from patients with CNS HSV infection; (d) three samples from patients with CNS HHV-6 infection, (e) eight samples from patients with CNS enterovirus infection; and (f) one sample from an untreated neurosyphilis patient. Also, the comparison sample panel included 34 samples from patients with confirmed (19) or suspected (8) multiple sclerosis (MS), or with other neuroinflammatory conditions (7). In all these 34 patients, CSF oligoclonal (OC) bands were detected.

### CXCL13 concentrations in CSF of different patient groups and in non-LNB samples

**Figure 2:**
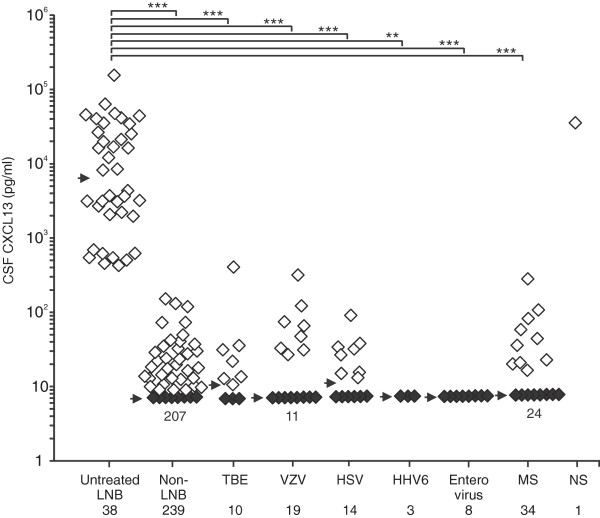
**CXCL13 concentrations in the LNB and comparison CSF samples.** Black diamonds indicate CSF samples with concentrations below the lowest standards of the assay (<7.8 pg/ml). If more than eight samples were below the lowest standard, the figure below the black diamonds indicates the number of such samples. The arrow indicates the median concentration. Kruskal-Wallis test followed by single pairwise comparisons with the Mann–Whitney *U* test and Bonferroni corrections were used for statistical analysis. **P < 0.01; ***P < 0.001.

The concentrations of CXCL13 in CSF of all untreated LNB patients ranged from 424 to 158,000 pg/ml, with the median of 6,480 pg/ml (Figure 2). Among the seven probable LNB cases, CSF CXCL13 concentrations ranged from 498 to 8,600 pg/ml, and among the non-LNB samples, from <7.8 to 153 pg/ml, with the median of <7.8 pg/ml. The difference in CSF CXCL13 concentrations between the untreated LNB patients and the non-LNB group was statistically significant (*P* < 0.001). Among the samples of the patients with viral CNS infections, the CSF CXCL13 concentrations ranged from <7.8 to 406 pg/ml, and the statistical difference between these viral CNS infection samples and the LNB samples was highly significant, with *P* values of 0.0013 or less in all cases. CSF CXCL13 concentrations in the 34 samples from patients with noninfectious neuroinflammatory conditions ranged from <7.8 to 280 pg/ml, which was also significantly different (*P* < 0.001) from the CXCL13 levels of LNB samples.

**Figure 3:**
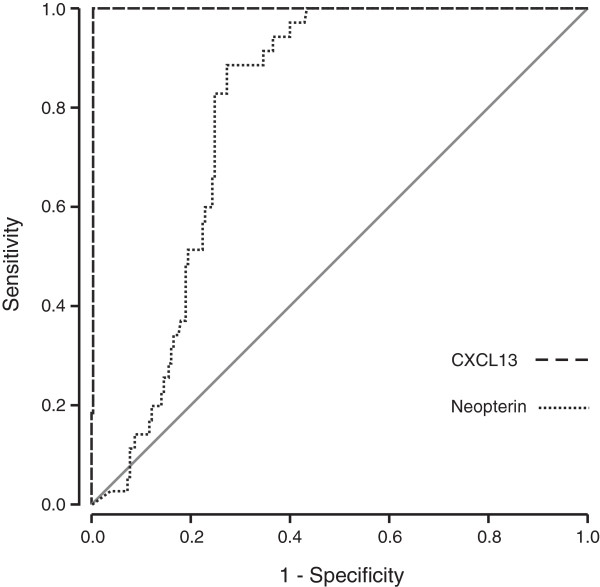
ROC curve analyses of CSF CXCL13 and neopterin levels were performed to obtain the best cut-off values that would discriminate between LNB patients and the comparison groups.

Neurosyphilis caused by the spirochete *Treponema pallidum* subsp. *pallidum* appears to be the only other disease among the conditions investigated in this study that leads to markedly increased CSF CXCL13 concentration. CXCL13 in the CSF sample of the single neurosyphilis patient was 37,000 pg/ml. These results clearly indicate that highly elevated CSF CXCL13 levels are associated with LNB, or with other spirochetal infections of the CNS.It is obvious from the receiver operating characteristic (ROC) curve analysis (LNB pretreatment samples against all comparison groups) shown in Figure 3 and also from the data presented in Figure 2 that the diagnostic performance of CSF CXCL13 determination with a cut-off 415 pg/ml results in nearly perfect discrimination between the LNB patients and other groups in this sample material. The use of this cut-off led to sensitivity of 100% and specificity of 99.7% for the diagnosis of LNB.

### Neopterin concentrations in CSF samples of the different groups

**Figure 4:**
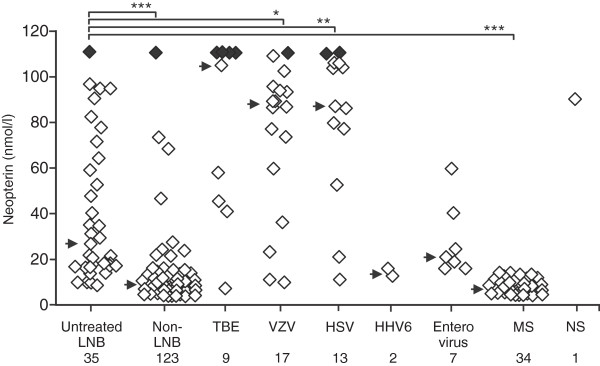
**Neopterin concentrations in the LNB and comparison CSF samples.** Black diamonds indicate CSF samples with concentration above the highest standards of the assay (>111 n*M*). The arrow indicates the median concentration. Kruskal-Wallis test followed by single pairwise comparisons with Mann–Whitney *U* test and Bonferroni corrections were used for statistical analysis. *P < 0.05; **P < 0.01; ***P < 0.001.

Neopterin concentrations were analyzed in the previously mentioned sample groups, although in most of the groups, the number of samples available for neopterin analysis was somewhat smaller than the number of samples in CXCL13 analysis (Figure 4). Neopterin concentration was significantly higher (*P* < 0.001) among the untreated LNB patients (median, 26.6 n*M*) than in the non-LNB group (median, 6.3 n*M*). Conversely, and in contrast to CSF CXCL13 levels, neopterin concentrations were widely scattered over the entire measuring range in LNB, TBE, VZV, and HSV samples. In fact, CSF neopterin levels were significantly higher among the VZV (*P* < 0.05) and HSV (*P* < 0.01) infected patients than among LNB patients. In the single neurosyphilis sample, the neopterin concentration was 92.2 n*M*. Among the samples of the other neuroinflammatory patients, CSF neopterin levels were uniformly low.ROC curve analysis (LNB pretreatment samples against all comparison groups) was performed also with the neopterin data, and the result is shown in Figure 3. The use of neopterin concentration 10.6 nM as the cut-off led to sensitivity of 88.6% and specificity of 65.0% for the diagnosis of LNB.

### Effect of antibiotic treatment on CXCL13 and neopterin concentrations in CSF of LNB patients

**Figure 5:**
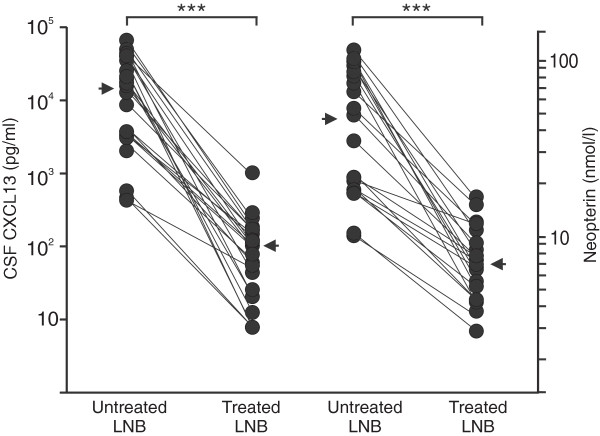
**CXCL13 (left) and neopterin (right) concentrations in CSF samples of LNB patients before and after antibiotic treatment.** Twenty four samples (21 for neopterin) of which 19 (16 for neopterin) were collected at the end of or right after the treatment, and five samples several months after the first sample were available for the analyses. The arrow indicates the median concentration. Wilcoxon signed-rank test was used to compare concentrations before and after treatment. ***P < 0.001.

Twenty-four posttreatment CSF samples were available for CXCL13 analysis from the confirmed LNB cases. Nineteen of the samples were collected at the end of or right after the antibiotic treatment. Five samples were collected several months after the first sample. The decline in CSF CXCL13 levels along the treatment was striking; the median concentration before the treatment among the 24 samples was 16,770 pg/ml (average, 19,730 pg/ml), and after the treatment, 109 pg/ml (average, 138 pg/ml) (Figure 5). The decline was statistically significant (*P* < 0.001). Among the five posttreatment samples that were collected only months after the treatment, CXCL13 was <7.8 pg/ml in four samples and 20.6 pg/ml in one sample.

Also, the CSF neopterin concentrations (21 posttreatment samples; 16 samples collected at the end of or right after the antibiotic treatment, five samples several months after the first sample) decreased statistically significantly along with the treatment (*P* < 0.001). The median concentration before the treatment was 47.9 n*M*(average, 51.1 n*M*) and after the treatment, 7.5 n*M* (average, 8.0 n*M*).

### Correlations among CSF parameters in LNB patients

**Figure 6:**
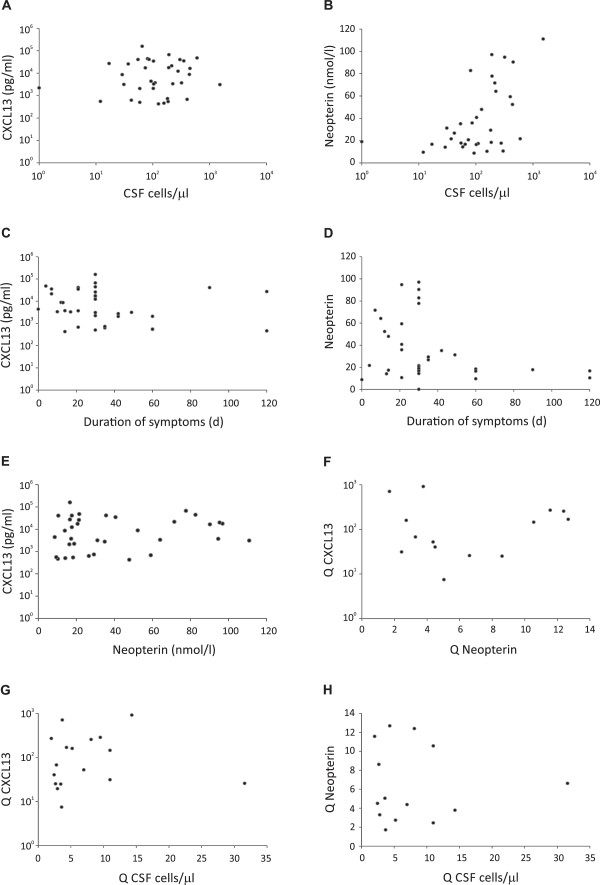
**Correlations among CSF parameters in LNB patients. (A)** CSF CXCL13 concentration in untreated LNB patients plotted against CSF cell count. **(B)** CSF neopterin concentration in untreated LNB patients plotted against CSF cell count. **(C)** CSF CXCL13 concentration in untreated LNB patients plotted against duration of symptoms before CSF sampling. **(D)** CSF neopterin concentration in untreated LNB patients plotted against duration of symptoms before CSF sampling. **(E)** CSF CXCL13 concentration in untreated LNB patients plotted against CSF neopterin concentration. **(F)** CSF CXCL13 quotient (see Statistical analyses for definition) plotted against neopterin quotient. **(G)** CSF quotient of mononuclear cells plotted against CXCL13 quotient. **(H)** CSF quotient of mononuclear cells plotted against neopterin quotient. No statistically significant correlations were observed.

The correlations of CSF parameters were analyzed among the pretreatment LNB CSF samples (Figure 6A-E). The correlations between the quotients of CSF mononuclear cells, CSF CXCL13, or CSF neopterin before and after treatment are shown in Figure 6F-H. No statistically significant correlation was observed among the different CSF parameters. In the analysis of CSF neopterin concentration versus CSF cell count (Figure 6B), a trend toward significant correlation was found, with *P* value 0.057.

## Discussion

The early diagnosis of LNB can be difficult because the intrathecal production of *Borrelia burgdorferi*-specific antibodies may be absent in the beginning of the disease [22]. The chemokine CXCL13 is a promising biomarker with a high sensitivity and specificity for early untreated LNB also in atypical cases [23]. Elevated concentrations of CXCL13 in the CSF may appear several days before intrathecal borrelial antibody synthesis occurs [10]. Intrathecal borrelia-specific antibody production may persist for years after successful treatment [4], whereas the levels of CXCL13 decrease rapidly after antibiotic treatment, and hence it might be suitable for monitoring of treatment response, and distinguishing an active infection from a past infection [24]. It has also been suggested that CSF CXCL13 may be a marker of disease duration in LNB rather than a marker of disease activity [7].

In the present study, the level of CSF chemokine CXCL13 was highly elevated in patients with untreated LNB and decreased rapidly after treatment. At the time of diagnosis, the levels of CXCL13 varied from 424 to 158,000 pg/ml (median, 6,480 pg/ml) in LNB patients. In non-LNB patients, the levels of CXCL13 varied from <7.8 to 153 pg/ml. The single patient with neurosyphilis had a CXCL13 level of 37,000 pg/ml. Neurosyphilis is known to elevate the level of CXCL13 in the CSF [7,8]. At the time of diagnosis, the levels of CXCL13 in the CSF were significantly higher in LNB patients than in comparison groups. The diagnostic sensitivity of CSF CXCL13 measurement was 100%, and specificity was 99.7% when using 415 pg/ml as the cut-off. Thus, our results strengthen previous findings that CXCL13 is an excellent tool for the diagnostics of early LNB.

Previous studies reported different cut-off values of CSF CXCL13 in LNB ranging from 61 pg/ml to 1,229 pg/ml [6-8,12,13]. The difference among cut-offs of these studies might reflect variability in the analytic test used for the chemokine measurement. However, in these studies, and in the present study, CXCL13 levels were measured by using the same commercial kit. The intercenter variability may also be due to, for example, variation in the comparison groups. When determining the sensitivity and specificity of a test, it is essential to use relevant comparison samples that is, in the case of LNB, comparison subjects should include patients with locally encountered diseases that cause symptoms similar to those caused by LNB. Thus, it is also advisable to use locally determined CSF CXCL13 cut-offs until a universally accepted cut-off level has been established.

The LNB patients were treated either with intravenous ceftriaxone, oral doxycycline, or with a combination of both, according to Finnish practices and international recommendations [25]. Levels of CSF CXCL13 decreased rapidly after antibiotic treatment. Nineteen of 24 posttreatment samples were collected 3 to 4 weeks after the first sample and institution of the antibiotic treatment. In five of 24 cases, the second CSF sample was taken 108 to 225 days after the first sample. The reason for the late control-sample collection was residual posttreatment symptoms. However, reinfections or relapses, as based on subjective symptoms, or serum or laboratory parameters, were not identified during the follow-up. Also in the late control-sample group, the levels of CXCL13 remained low. It remains to be investigated whether CSF CXCL13 levels increase again after a reinfection or relapse. Also, additional studies are needed to correlate the change in CSF CXCL13 levels with subjective and objective recovery after LNB treatment.

Elevation of CSF neopterin is a marker of an acute or active CNS inflammation, but it is not specific to any particular disease [26]. Neopterin has been suggested as a useful marker for the evaluation of the posttherapeutic outcome in patients with sleeping sickness caused by *Trypanosoma brucei gambiense* infection [27]. In our study, the levels of neopterin were high in a number of CSF samples, including those obtained from LNB patients, but also in samples from patients with viral (especially TBE, VZV, and HSV) CNS infections. However, low CSF neopterin concentrations were also observed in the same groups. Thus, neopterin is neither a sensitive nor a specific marker in these conditions. The levels of CSF neopterin decreased after antibiotic treatment in LNB patients. We conclude that because of the low sensitivity and specificity, neopterin measurements cannot be recommended as a tool for purposes of diagnosis of LNB or for assessment of LNB treatment outcome. Because CSF neopterin levels were uniformly low in the samples of the patients with MS or other neuroinflammatory diseases, CSF neopterin may in some cases help in distinguishing neuroinflammatory conditions from neuroinfection.

In statistical analyses, no significant correlations were found between different CSF parameters in the pretreatment LNB samples, or in the quotients of CSF mononuclear cells, CXCL13, or neopterin before and after the treatment. Only in the correlation of CSF neopterin concentration versus CSF cell count was a trend noted toward significance with *P* value 0.057. In a previous study, a significant correlation occurred between CSF mononuclear cells and CXCL13 [13]. The reason for this correlation remains unclear.

Some limitations exist in the current study. First, because of the nature of the study, the samples were mainly from preselected hospital patients, and no CSF samples of healthy people were included. The borrelia antibody-negative non-LNB samples were collected during diagnostic workup from patients with some degree of neurologic symptoms, and thus they do not represent healthy people. Also, the untreated LNB patients were older (median age, 66 years) than non-LNB subjects (median age, 50 years). However, CSF CXCL13 concentrations among these non-LNB samples were uniformly low, highlighting the specificity of elevated CSF CXCL13 as a biomarker for LNB.

Second, seven patients represented probable LNB cases based on normal or not determined AI, or on lack of CSF pleocytosis. However, all these patients did have antibodies against *Borrelia burgdorferi* in the CSF and serum, and their CSF CXCL13 concentrations ranged from 498 to 8,600 pg/ml, suggesting also that these patients were most likely true LNB patients. Also, the clinical manifestations among the probable LNB patients (radiculitis and/or facial nerve paralysis) fit in with the diagnosis of LNB. No follow-up samples were available of the probable LNB patients, who had normal AI in the pretreatment CSF sample, and thus it remains unsolved whether the AI would have turned positive later during the infection.

Third, sample storage at −20°C before testing may in principle decrease levels of CXCL13, and thus result in falsely low concentrations [12]. Some of the LNB patient samples and all the comparison samples had been stored frozen before the cytokine analyses. In one study, the duration of storage of a sample did not result in lower levels of CXCL13 in the LNB group, as the concentration of CXCL13 did not decrease during storage for up to 5 years [8]. We also performed a control assay in which one CSF sample was divided in five aliquots that were frozen and thawed up to 5 times. The procedure did not influence the levels of CXCL13 (Hytönen *et al*. unpublished data).

Fourth, no CSF samples of patients with purulent meningitis, CNS lymphoma, cryptococcosis, African trypanosomiasis, or HIV were among the comparison samples. Previously, it was reported that CSF CXCL13 may be elevated in patients with these conditions [6,8,13,28]. However, we selected the comparison samples especially from patients who had diseases that are potential differential diagnostic options for LNB in Finland. Meningitis caused by typical bacteria is usually easy to discriminate from LNB because of the clinical presentation, serum inflammatory markers and CSF white cell count, granulocytic differential count, microscopy, and bacterial culture. Thus, purulent meningitis samples were not studied. Samples from CNS lymphoma and neurologically symptomatic HIV patients were not available.

## Conclusions

The results of the present study suggest that CSF neopterin is an insensitive and unspecific biomarker that is not suitable for diagnostics of LNB, but it may have role in distinguishing neuroinfective diseases from neuroinflammatory states. In contrast, CSF CXCL13 appears to be an excellent biomarker in differentiating LNB from viral CNS infections and from other neuroinflammatory conditions when locally determined cut-offs are used. It is also a useful tool for follow-up of LNB patients after antibiotic treatment. However, CSF CXCL13 results should be interpreted in conjunction with intrathecal borrelia antibody production, CSF pleocytosis, and ideally also borrelia nucleic acid amplification result. Limited information is available of CSF CXCL13 levels in chronic LNB [7], or in relapses and reinfections. Further studies are required to correlate the decline in CSF CXCL13 levels with clinical recovery after LNB treatment.

## Abbreviations

AI: Antibody index; CNS: central nervous system; CSF: cerebrospinal fluid; CXCL13: chemokine (C-X-C motif) ligand 13; CXCR5: chemokine (C-X-C motif) receptor 5; HHV-6: human herpesvirus 6; HIV: human immunodeficiency virus; HSV: herpes simplex virus; LNB: Lyme neuroborreliosis; MS: multiple sclerosis; NS: neurosyphilis; OC: oligoclonal; ROC: receiver operating characteristic; TBE: tick-borne encephalitis; TPHA: *Treponema pallidum* hemagglutination assay; VDRL: Veneral Disease Research Laboratory; VZV: varicella zoster virus.

## Competing interests

The authors declare that they have no competing interests.

## Authors’ contributions

JH designed the study, supervised the chemokine analyses, analyzed the data, and drafted the manuscript. EK and JO participated in the design of the study, collected the clinical data, and helped to draft the manuscript. JP helped to interpret the data and to draft the manuscript. MW was responsible for the viral analyses and drafted parts of the manuscript. JS performed the statistical analyses and drafted parts of the manuscript. All authors read and approved the final manuscript.
